# *In vitro* and *in vivo* characterization of Entacapone-loaded nanostructured lipid carriers developed by quality-by-design approach

**DOI:** 10.1080/10717544.2022.2058651

**Published:** 2022-04-05

**Authors:** Yogeeta Agrawal, Kiran Patil, Hitendra Mahajan, Mrugendra Potdar, Pratiksha Joshi, Kartik Nakhate, Charu Sharma, Sameer N. Goyal, Shreesh Ojha

**Affiliations:** a Department of Pharmaceutics, Shri Vile Parle Kelavani Mandal’s Institute of Pharmacy, Dhule, India; b Department of Pharmaceutics, R. C. Patel Institute of Pharmaceutical Education and Research, Shirpur, India; c Department of Pharmacology, Shri Vile Parle Kelavani Mandal’s Institute of Pharmacy, Dhule, India; d Department of Internal Medicine, College of Medicine and Health Sciences, United Arab Emirates University, Al-Ain, United Arab Emirates; e Department of Pharmacology and Therapeutics, College of Medicine and Health Sciences, United Arab Emirates University, Al Ain, Abu Dhabi, United Arab Emirates

**Keywords:** Parkinson's disease, Entacapone, quality by design, nanostructured lipid carriers

## Abstract

Entacapone, a reversible catechol-o-methyl transferase inhibitor, is used to enhance the action of dopamine agonists by reducing their metabolism and the ‘Wearing-off’ effects associated with long-term use in the treatment of Parkinson's disease. It is used as an adjunct to levodopa/Carbidopa therapy. Due to limited dissolution and first-pass clearance, it suffers low and variable bioavailability issues. To overcome this problem, the present study aims to explore the potential of nanostructured lipid carriers (NLCs) for the delivery of Entacapone. The Quality by Design (QbD) approach was used for the systematic development of NLCs. The 2^3^ full factorial designs were investigated using Design-Expert®11 software. The three independent variables namely content of total lipid (X1), surfactant (X2), and sonication time (X3) were optimized against two responses namely particle size and entrapment efficiency. The optimized NLCs were characterized for their size, surface morphology, entrapment efficiency, drug release, thermal and crystallographic studies. In-vivo pharmacokinetic studies in Entacapone-loaded NLCs showed an increase in *t*_1/2_, AUC_0–∞_, MRT compared to free drug. The reduction in elimination (Kel) depicts the prolonged action of Entacapone by loading in NLCs. The results displayed Entacapone-loaded NLCs have promising potential for oral delivery and enhanced therapeutic effect which otherwise was a major issue.

## Introduction

Parkinson’s disease (PD) is a neurodegenerative disorder affecting 7–10 million people worldwide. It is caused due to the impairment of small areas in the brain that control posture, balance, and movement (DeMaagd & Philip, 2015; Dorsey et al., 2018). There is no perfect treatment available for PD; however, therapy aims to minimize the impact of symptoms. The symptomatic treatment involves dopamine agonists like levodopa and MAO-B inhibitors. However, conventional treatment becomes less effective as the disease worsens and produces end-of-dose adverse effects known as ‘wear off’ symptoms. These symptoms are characterized by the reappearance of both motor and non-motor movements of PD as a result of previous carbidopa-levodopa therapy (Chahine et al., 2020).

**Figure 1.**
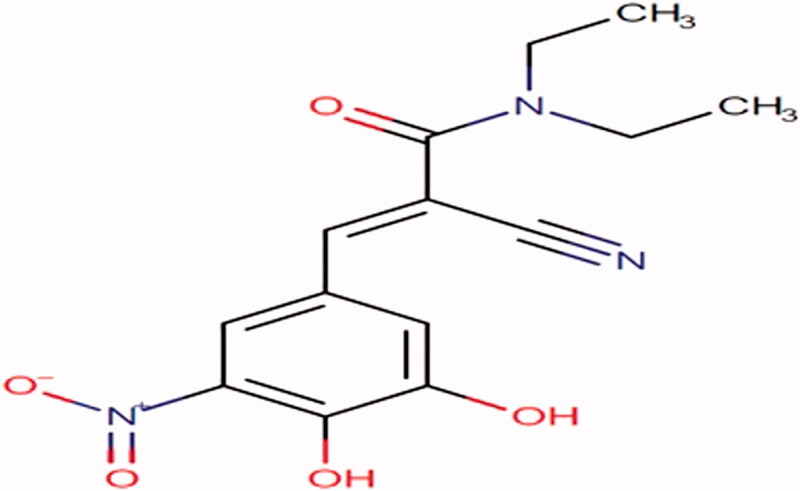
Structure of Entacapone.

Entacapone, a nitro catechol compound (Figure 1), has been approved for clinical use in patients with PD. It inhibits the degradation of dopamine and levodopa by blocking the enzyme Catechol-O-Methyl Transferase (COMT) (Najib, 2001). It enhances the action of dopamine and reduces the onset of motor complications to a certain extent. It is used along with carbidopa-levodopa therapy to overcome the ‘wear-off’ symptoms (Antonini et al., 2018; Müller, 2020). But Entacapone is a BCS class IV drug with low aqueous solubility and low permeability (Bommaka et al., 2018). Moreover, the bioavailability may also be affected by high lipophilicity, pre-systemic clearance in the gastrointestinal mucosa, and the P-GP efflux mechanism (Garg et al., 2020). Therefore, the major challenge is to formulate the drug delivery system that possibly tackles all these problems and increases the bioavailability and residence of the drug.

Several technologies can be used to enhance the dissolution characteristic of such low solubility and low permeability drugs. A lipid-based formulation is an alternative approach for developing a product from laboratory scale to commercial level successfully (Wen et al., 2015). Lipid-based gallows are potential drug carriers due to their propensity to improve the solubility of lipophilic drugs and ultimately augment oral bioavailability (Ashkar et al., 2021).

Nanostructured lipid carriers (NLCs) are novel lipid-based drug-delivery carriers comprised of solid and liquid lipids as a core matrix. These are second-generation lipoidal carriers that can overcome the limitations of first-generation solid lipid nanoparticles (SLNs), such as inefficient drug loading, drug leakage, polymorphism, and irrelevant gelation. The oil phase (liquid lipids) produces imperfections in the solid lipid, which increases carriers’ structural fluidity and results in high drug loading (Gordillo-Galeano & Mora-Huertas, 2018; Ashkar et al., 2021). Moreover, the use of affordable, biodegradable, and biocompatible lipids and ease of formulation with high stability made this delivery system an attractive promising alternative to first-generation nano-metric systems. NLCs have much justified to overcome the basic shortcomings of other Nano-carriers and are chosen for their known benefits like easy scale-up, high stability, high drug loading and minimum drug leakage, and prolonged drug release. Additionally, it has been reported that NLCs, in the intestinal lumen are exposed to lipases and co-lipase enzymes and are transformed into mixed micelles which are readily absorbed. Moreover, the drug is transported through the lymphatic route or payer’s patches, which eventually enhances oral absorption (Gordillo-Galeano & Mora-Huertas, 2018).

The possibility of formulation and development of Entacapone NLCs via optimization and analysis by *in vitro* and *in vivo* studies has not been explored yet, leading us to current research work. So the primary aim was to explore the potential of NLCs for the delivery of Entacapone in the management of Parkinson’s disease using the Quality by Design (QbD) approach. The secondary objective was to characterize it by Fourier transform infrared spectroscopy (FTIR), differential scanning calorimetry (DSC), powder X-ray diffraction (PXRD), scanning electron microscopy (SEM), drug particle size, zeta potential, entrapment efficiency, and stability study. And finally, to evaluate it by in-vivo pharmacokinetic studies in Wistar rats.

## Materials and methods

### Materials

Entacapone was obtained as a generous gift from Macloeds Pharmaceuticals Ltd., Mumbai, India. Glycerol monostearate (GMS), Oleic acid, and Tween 80 were received as a gift sample from S.D. Finechem. Ltd. Mumbai, India. Hydrogenated palm oil, Olive oil, were obtained as gift samples from Ultima Chemicals Limited, Gujarat, India. All the chemicals such as trehalose, acetonitrile, ethanol, methanol, and trimethylamine were of analytical grade and purchased from Merck Specialtie Pvt. Ltd., Mumbai, India.

### Screening of lipids for drug solubility

The preliminary requirement of NLCs is to investigate the various lipids for drug solubility and compatibility. The screening was based upon the maximum drug that could be solubilized in the suitable lipids. In the present study, various liquid lipids (Glycerylcaprate, Hydrogenated palm oil, Olive oil) and solid lipids (Glycerol monostearate, Glyceryl tribehenate, Glyceryl palmitostearate) were investigated.

The solubility of Entacapone in liquid lipids was investigated as per the previously explored method (Agrawal et al., 2021). Entacapone was added in increments with 2 mL of the investigational oil, and 3 mL of ethanol until a homogeneous mixture was obtained. The mixture was centrifuged at 25,000 rpm for 20 min. The clear supernatant obtained was suitably diluted and analyzed spectrophotometrically for Entacapone at 306 nm. The liquid oil that dissolved the highest amount of drug was selected.

For the solubility in solid lipids, drug was added in increments to 1 g of accurately weighed solid lipid and the test tube was heated (80 °C) on a water bath with continuous stirring (300 RPM) till the clear melt was achieved. The amount of lipid required to solubilize the complete drug was estimated.

### Preparation of Entacapone-loaded NLCs

Entacapone-loaded NLCs were prepared using the probe sonication method described by Agrawal et al. (2021). For the study, solid and liquid lipids were taken in a constant ratio (70:30). The melted lipid phase containing the drug was dispersed in an aqueous surfactant solution heated at the same temperature (80 °C) and stirred at 1800 rpm (Remi Instruments Ltd., Mumbai, India) to produce a pre-emulsion. This warm pre-emulsion was sonicated for 10 min with a probe sonicator (Sonics & Materials, Inc., Newtown, CT, USA) at 60% amplitude on 20 s pulse and 10-s rest cycle to form the NLC’s dispersion. Then the NLCs so formulated were allowed to be cooled at room temperature, which was further used for characterization.

### Optimization by quality by design

The QbD approach was used to optimize the developed NLCs. The 2^3^ full factorial design was investigated using Design-Expert^®^11 software (Stat-Ease, Inc., Minneapolis, MN, USA) (Ajiboye et al., 2021). The three independent variables namely content of total lipid (X1), surfactant (X2) and sonication time (X3) were optimized against two responses namley particle size and entrapment efficiency. Each response coefficient was studied for statistical significance at the 95% confidence level. A *p* > .05 value depicted that the model was significantly fit for the study. All the independent variables, their levels as well as the actual and coded values of these variables are given in (Table 1). The effect of the variables on the responses was statistically interpreted by the Fishers ANOVA test. The model suitability was checked by comparing adjusted and regular correlation coefficients (*r*^2^). Moreover, the simultaneous interaction of all variables on the responses was interpreted graphically with the 2D counter plot and 3D surface response plots. For optimization, the constraint was applied as minimum particle size, maximum entrapment efficiency (%), and minimum polydispersity index.

**Table 1.** 2^3^ Factorial design for Entacapone-loaded NLCs.

Run	X1 (g)	X2 (%)	X3 (min)
F1	0.5	1	13
F2	1	2	15
F3	0.5	2	15
F4	0.5	1	13
F5	1	2	13
F6	1	1	15
F7	1	1	13
F8	0.50	1	13

### Lyophilization of Entacapone NLCs

Trehalose (2%) was added to the aqueous dispersion of NLCs and frozen at −75 °C in deep freeze for 76 h. The frozen NLCs were then lyophilized by a freeze dryer (VirTis Benchtop K, SP Scientific, Warminster, PA, USA) and stored in sealed vials for further studies (Panda et al., 2020).

### Characterization of nanoparticles

#### Determination of particle size and PDI of prepared NLCs

The average particle size, polydispersity index (PDI), and surface charge of the NLCs was measured using a dynamic light-scattering method (Zetasizer Nano ZS, Malvern, UK) (Khan et al., 2019). It measures statistical fluctuations in the intensity of light scattered by particles caused by random Brownian motion. The NLCs suspension was dispersed uniformly in double-distilled water (1:100) and screened for particle size at varying scattering intensity (Patil and Deshpande, 2021).

#### Surface morphology

The surface morphology and the particle size of the formed NLCs were depicted using scanning electron microscopy (SEM, Zeiss, Jena, Germany). The gold coating of an aqueous dispersion of the NLCs was done by a dual ion beam sputtering system. The gold-coated droplets were then placed through accelerating voltage of 5.0 kV and magnification of 10–20 KX at 25 ± 2 °C temperature. The image of the NLCs was then captured at various magnifications as required (Patil et al., 2020).

#### Encapsulation efficiency

The percent encapsulation efficiency (%EE) of Entacapone in NLCs was estimated using the process described by Patil et al. (2020). The suspension of Entacapone-NLCs was centrifuged at 20,000 rpm and the concentration of unentrapped drug in the supernatant was estimated using UV–visible spectrophotometer at 306 nm *λ*_max_. Values of EE% were calculated using the following equation:

$$\%EE=\frac{\text{Initial drug in NLCs }-\text{ amount of unbound drug}}{\text{Initial drug in NLCs}}\;\times \;100$$

#### Differential scanning calorimetry (DSC)

DSC is a basic way to investigate the crystallinity or amorphous state of drugs, polymers, and formulations by determining the temperature and energy divergence at the phase transition. The thermal behavior of the Entacapone, bulk GMS, physical mixture of Entacapone with GMS in 1:1 ratio, and lyophilized Entacapone-NLCs were evaluated by DSC calorimeter (DSC STAR System, Mettler-Toledo, India Pvt. Ltd., Chennai, India). This study helps interpret the physiochemical changes in the samples concerning temperature. Moreover, it also provides vital information regarding drug and excipient compatibility. The samples were kept under constant heating rate with an increment of 10 °C/min for a temperature range of 25–300 °C under nitrogen purge, and the resultant thermogram was recorded.

#### X-ray diffraction

The crystalline nature of the NLCs was evaluated by an X-ray diffractometer (Bruker AXS D8 Advance, Karlsruhe, Germany). The samples of pure Entacapone, a physical mixture in (1:1) ratio, and lyophilized NLCs were screened for XRD patterns in the 3 to 80-degree range with a chart speed of 5 per minute.

#### Accelerated stability studies

Entacapone–NLCs were subjected to accelerated stability studies (Patel et al., 2012). The optimized batch of lyophilized drug-loaded NLCs was kept for accelerated stability study according to ICH Q1A (R2) guidelines (Trimukhe et al., 2019). The guideline suggests long-term stability at 5 °C ± 3 °C and accelerated stability at 25 °C ± 2 °C/60% RH ± 5% RH for 3 months for products to be stored in refrigerators (Muthu Madaswamy, 2009). The stability of the NLCs was checked concerning particle size, PDI, zeta potential, and entrapment efficiency.

#### In vitro release studies of entacapone NLCs

Entacapone–NLCs equivalent to 10 mg of drug were placed in a dialysis bag with a pore size of 2.4 nm and molecular weight cut off between 100 kDa. The dialysis bags were tied at both ends and were placed in the beaker containing 250 mL hydrochloric acid (0.1 N) for an initial 2 h. Then the media was replaced with phosphate buffer saline pH 6.8. The beaker was maintained at 37 ± 2 °C and stirred at a constant speed of 100 rpm [ICH, XXXX]. At regular intervals of 0, 0.5, 1, 1.5, 2, 3, 4, 5, 6, 7, 8, 9, 10, 11, 12, 18 and 24 h, 500 µL of dissolution medium was removed and was replaced with the fresh buffer at the same temperature to maintain sink condition. The amount of Entacapone in the aliquots was analyzed by UV–visible spectrophotometer at max 306 nm (Muthu Madaswamy, 2009).

#### Pharmacokinetic study

To study the fate of Entacapone–NLCs, the *in vivo* pharmacokinetic study was carried out for optimized formulation and plain drug suspension as per the below-discussed protocol. The experimental protocol in the present study was approved by the Committee for Control and Supervision of Experiments on Animals (CPCSEA) and the Institutional Animal Ethics Committee (IAEC) of R.C. Patel Institute of Pharmaceutical Education and Research, India (Protocol No. RCPIPER/IAEC/201).

#### Experimental design

The experiment was carried out on healthy adult Sprague–Dawley rats with a weight range from 200 to 250 g. The animals were housed in acrylic cages (45 × 24 × 20 cm), maintained at constant room temperature (25 ± 2 °C), relative humidity (50 ± 5%) and 12-h dark/light cycle with ad libitum access to pelleted chow food and drinking water (Chalikwar et al., 2012; D’Souza, 2014). Before dosing, the animals fasted for the period of 12 h prior and 4 h post with free access to water. Animals were divided into two groups (*n* = 6 per group). The control group received a suspension of free Entacapone suspended in 0.5% *w/v* sodium CMC (Gautam et al., 2019) and the test group received the drug-loaded optimized formulation at a dose of 3 mg/kg body weight (Forsberg et al., 2003; Bampidis et al., 2020; Patil et al., 2020).

#### Pharmacokinetic data analysis

The blood samples were collected in an aliquot (0.5 mL) by the retro-orbital plexus of each animal at the scheduled time intervals namely, 0.5, 1, 2, 4, 8, 12, and 24 h. The serum concentration versus time profile for the drug and formulations were estimated from the non-compartmental pharmacokinetic model, and the parameters were evaluated by Kinetica software, 5.0 (Thermo Fisher Scientific, Waltham, MA). The PK parameters such as peak plasma concentration (*C*_max_), time to reach *C*_max_ (*T*_max_), AUC_0–∞_, mean residence time (MRT) and half-life were calculated using the concentration–time plot of the drug.

#### Sample treatment procedure

The Eppendorf tube consisting of an aforementioned mixture was meticulously vortex-mixed (Macro Scientific Work Pvt Ltd, Delhi, India) for 30 s followed by centrifugation at 15,000 rpm for 10 min at 6 °C to separate denatured protein. After centrifugation, 20 µL of the filtered supernatant (0.45 lm membrane filter) was injected into the HPLC system and analyzed at 306 nm using Promethazine as an internal standard (Chalikwar et al., 2012; Gonçalves et al., 2012).

## Results and discussion

### Screening of lipids for drug solubility

Solubility of Entacapone in different solid lipid and liquid lipids were estimated. The results indicate that the relative distribution of Entacapone in GMS was higher 42.01 ± 0.05 mg than the other solid lipids. Whereas, Oleic acid has higher solubility 47.71 ± 0.77 mg of the drug compared to the other liquid lipids. Therefore, GMS and Oleic acid were chosen as the solid and liquid lipid for the formulation of Entacapone-loaded nanostructured lipid carriers (NLCs) respectively.

### Preparation of Entacapone-loaded NLCs

The probe sonication technique was used to prepare Entacapone-loaded NLCs. This technique produces smaller NLCs with minimum poly-dispersity and high drug loading. Moreover, it is reproducible, robust and easy for large-scale production. The GMS and Oleic acid were chosen as solid lipid:liquid lipid (70:30), respectively. Based on the results, it was suggested that Tween 80 was sufficient to effectively cover 1% of the nanoparticle surface and prevent agglomeration during the homogenization process. Lipid nanoparticles stabilized with the Tween 80 have low particle size and high storage stability.

### Optimization of NLCs by quality by design

After selecting the most important factors affecting Entacapone NLCs, 2^3^ simple full factorial design was used to determine optimal levels of these variables. Table 2 depicts the experimental results for the responses such as the particle size (Y1) and drug entrapment efficiency (Y2) of the NLCs. The particle size of all batches was between 161 and 240 nm. The entrapment efficiency of all the batches was found to be in the range of 73–82%. A mathematical relationship between factors and parameters was generated by factorial design analysis using Design-Expert software.

**Table 2.** Formulations batches of Entacapone–NLCs.

Batch	X1	X2	X3	Y1	Y2
F1	0.50	1.00	13	240	79
F2	1.00	2.00	15	161.2	82.5
F3	0.50	2.00	15	213.4	75
F4	0.50	1.00	13	225.2	80
F5	1.00	2.00	13	230.5	78
F6	1.00	1.00	15	206.4	73
F7	1.00	1.00	15	218.4	74
F8	0.50	1.00	13	220.8	76

The statistical significance and the interaction of each variable were estimated using different polynomial models. It was observed that the quadratic polynomial model was accurate to predict and interpret the relationship between the CQAs with the given responses. This was also confirmed with the *F*-test ANOVA (*p* < .05) value which depicts the significance of the model. Table 3 summarizes all the results of the full factorial design. The comparable high correlation coefficient (*R*^2^) values of all the responses signify the best fit of the quadratic polynomial model. The insignificant (*p* > .05) lack of fit values represents the pure error which also confirms the best fit of the model. Moreover, the difference between predicted *R*^2^ and adjusted *R*^2^ values concerning actual *R*^2^ value is less than 0.2 which signifies the accuracy of the selected model.

**Table 3.** Statistical report of critical quality attributes using full factorial design.

Models	Model Fit		Actual *R*^2^	Adjusted *R*^2^	Predicted *R*^2^	S.D.	% C.V.
	*F* value	*p* Value					
Particle Size (Y1)	339.16	.0415	0.9995	0.9966	0.9686	10.04	3.23
% EE (Y2)	258.33	.0476	0.9994	0.9955	0.9587	0.35	0.39

The coefficients of regression equations for fitted quadratic model:

Particle size=+31.15+153.23X1−41.63X2−18.85X3+0.70X1X2−6.93X1X3+5.58X2X3

EE=+90.63+3.37X1+2.62X2+1.87X3−0.62X1X2−0.87X1X3−1.12X2X3

where X1, X2, and X3 are coded values for independent variables for the concentration of total lipid, the concentration of surfactant, and sonication time, respectively.

**Figure 2.**
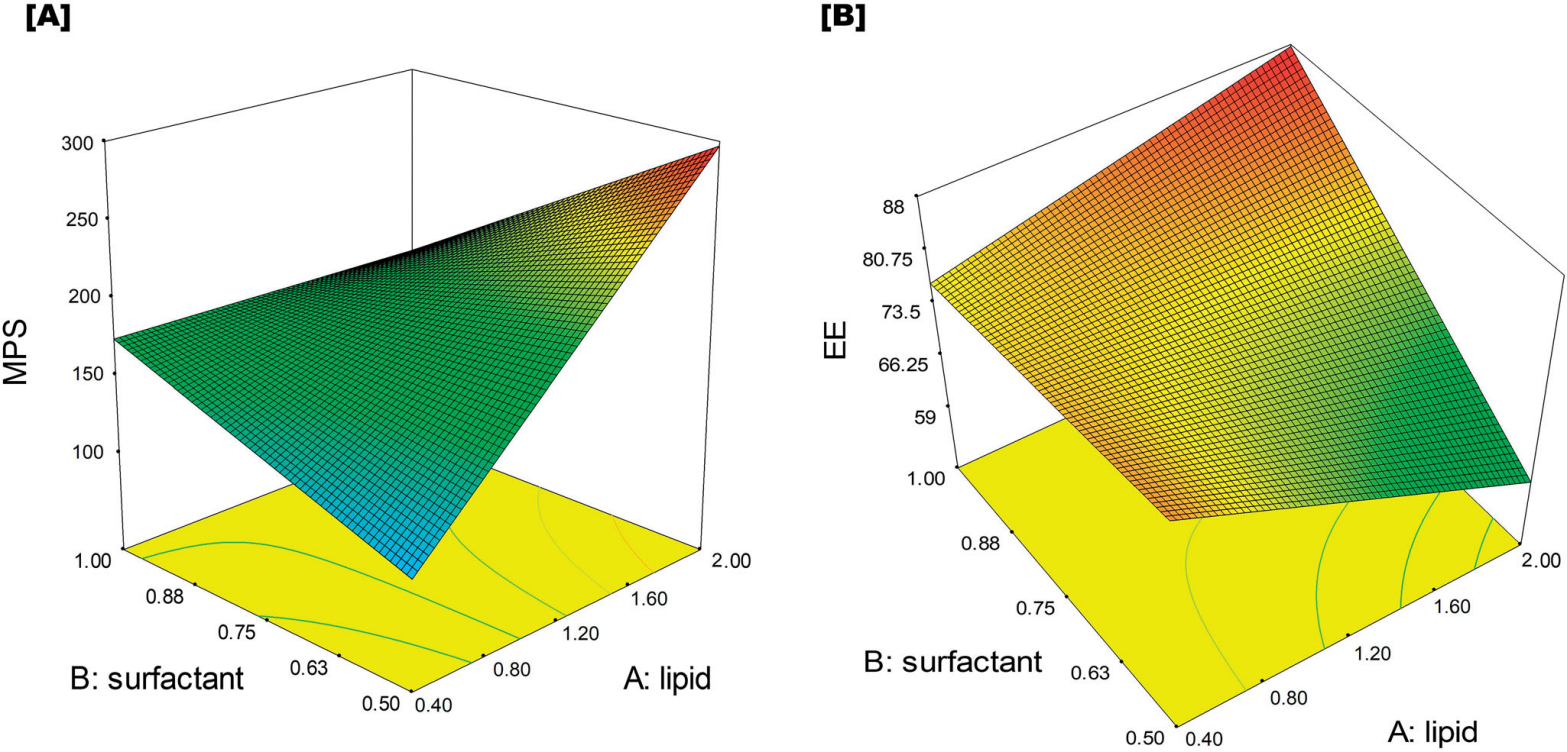
Response surface plot showing the effect of variables i.e. total lipid concentration and surfactant concentration over the particle size [A] and entrapment efficiency [B].

To study the simultaneous interaction of all the CQAs, three-dimensional (3D) response surface plots (Figure 2) were generated and studied. It was observed that total lipid concentration was directly proportional to the particle size of the NLCs and increased when the concentration of lipid increased. However, surfactant concentration was inversely proportional to the particle size of the NLCs. Moreover, sonication time has also pronounced effect on particle size and was inversely proportional to each other. This might be due to the higher molecular clouding at high lipid concentration which leads to agglomeration of NLCs. In contrast at higher concentrations of surfactant sufficient charges were developed on the surface of NLCs which reduces the agglomeration and stabilizes the formed NLCs. While at higher sonication time, NLCs break down to smaller size. Consequently, all three CQAs significantly affect the EE and it increases with an increase in total lipid concentration and surfactant concentration while reducing with an increase in sonication time. The better availability of lipid and surfactant increases the drug dissolution and entrapment capacity of the NLCs. While sonication tends to leach out the drug from formed NLCs. The observation was well with previously reported results of particle size and EE (Kaakkola, 2010; Gonçalves et al., 2012; Mahmood et al., 2021). Therefore, optimal responses could be achieved when the variable amount was the total lipid concentration (X1) 0.5%, surfactant concentration (X2) 1–2% and 13–15 min of sonication time (X3).

### Characterization of NLCS

#### Particle size, poly-dispersity index, and zeta potential

**Figure 3.**
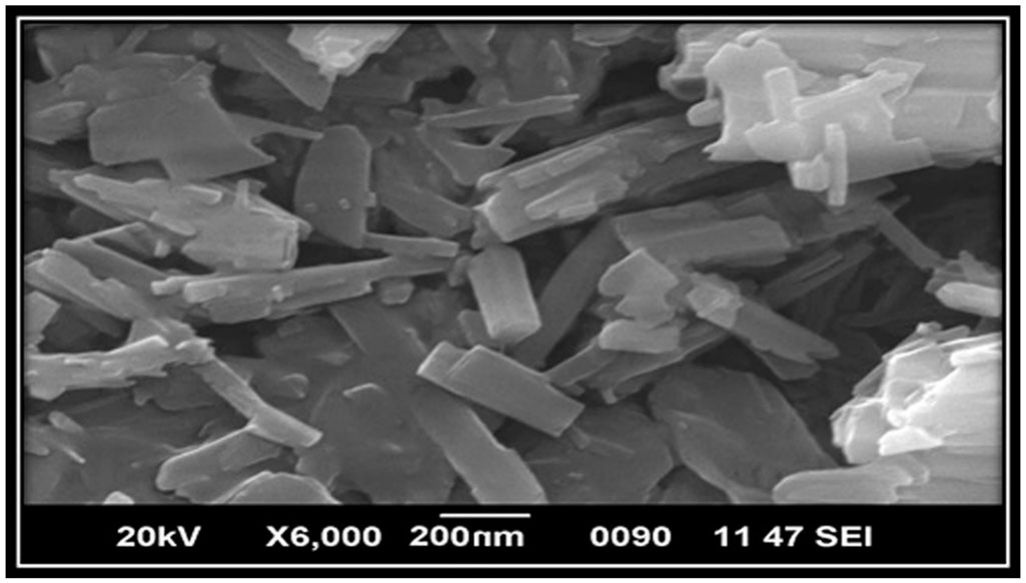
Scanning electron microscopic (SEM) photograph of Entacapone-loaded NLCs.

**Figure 4.**
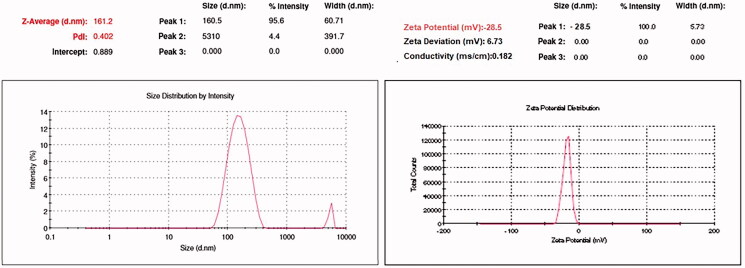
Particle size and zeta potential of Entacapone-loaded NLCs.

The morphology of the optimized batch was studied with the help of scanning electron microscopy (SEM) and confirms the non-spherical shape and nano-metric size of the drug-loaded NLCs (Figure 3). This was also confirmed by the measurement of particle size, PDI, and zeta potential using Malvern Zeta Sizer and found to be 161.2 ± 16 nm, 0.444, and −28.5 mV, respectively (Figure 4). The negative zeta potentials were likely due to the negatively charged lipid (GMS) and surfactants (Tween 80).

#### Entrapment efficiency

The entrapment efficiency of the optimized batch of drug-loaded NLCs was found to be 82.5 ± 3%. The high entrapment was likely due to the increased percentage of liquid lipid (oleic acid) which has greater solubility of the drug and the less ordered solid lipid matrix.

#### Differential scanning calorimetry

**Figure 5.**
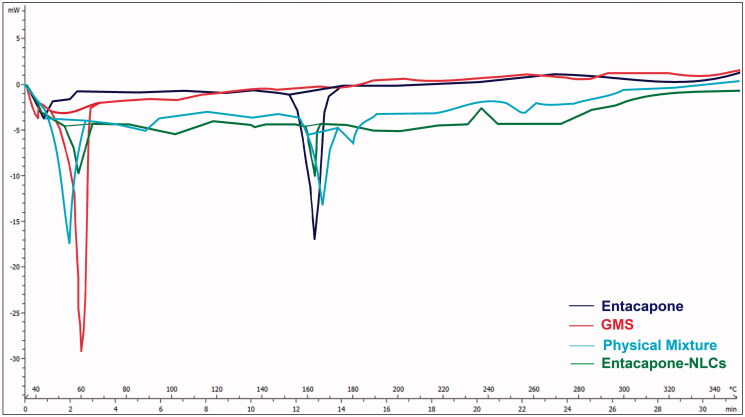
DSC overlay thermogram of Entacapone, GMS, physical mixture, and Entacapone NLCs.

Differential scanning calorimetry (DSC) studies were conducted for Entacapone, bulk GMS, their physical mixture in 1:1 ratio and freeze-dried NLCs of the optimized batch and shown in Figure 5. The overlay of the DSC thermogram shows the sharp endothermic peak of free Entacapone at 163.6 °C and endothermic peak for GMS at 61.57 °C. Distinct peaks of both GMS and Entacapone are observed in the graph obtained with represents no chemical interaction. The DSC thermogram of lyophilized NLCs showed one endothermic peak at 61.49 °C due to the existence of a lipidic phase and a very small peak at 163 °C. The reduced intensity of peak was due to the molecular incorporation of Entacapone into the lipid matrix of the GMS. This suggests that the Entacapone exists in the amorphous state in the formulation and is homogeneously dispersed in the NLCs. Thus, the DSC study concluded the absence of any chemical interaction between Entacapone and the lipidic phase. A lowering of the melting point of the lipid matrix as compared to their stuffing was observed which may be due to the presence of tween 80.

#### X-ray diffraction

**Figure 6.**
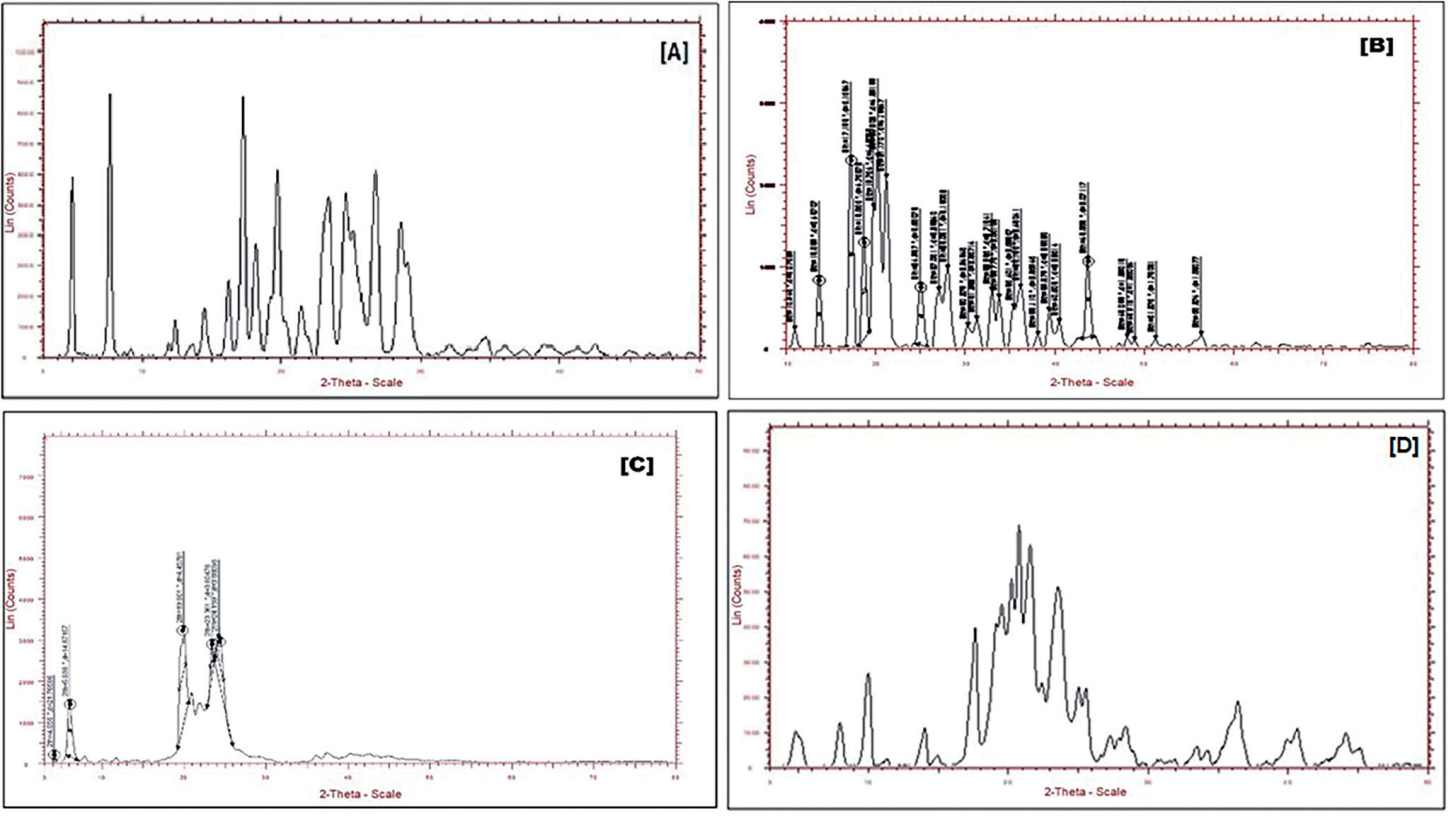
XRD of plain Entacapone [A] and GMS [B], Physical mixture [C], and Lyophilized Entacapone–NLCs [D].

X-ray diffraction pattern (XRD) studies of the purified drug Entacapone, GMS, physical mixture and lyophilized Entacapone nanostructured lipid carrier (NLCs) formulation were performed using an X-ray diffractometer. The 2*Ɵ* value of Entacapone as a pure drug showed sharp diffraction peaks which reflects the crystalline nature of pure Entacapone. However, the physical mixture and NLCs show the amorphous nature in the diffractogram indicating that the new structure has not been formed. This may be due to the molecular entrapment of the drug in NLCs. Similar studies were also reported by various formulation scientists (Kaakkola, 2010; Gonçalves et al., 2012). The X-ray diffraction patterns of Entacapone and freeze-dried NLCs preparation are given in Figure 6.

#### *In-vitro* Drug Release

**Figure 7.**
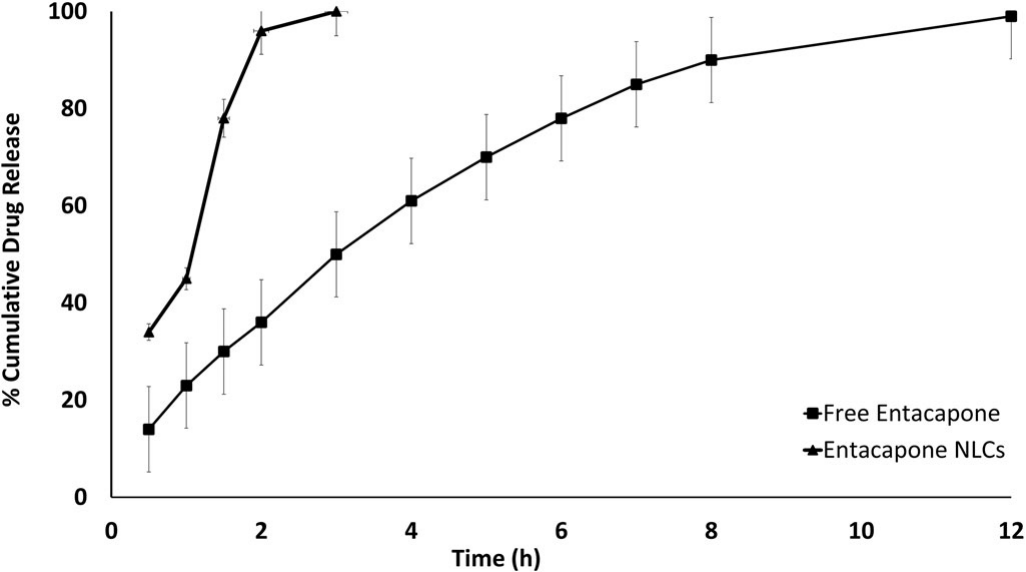
*In vitro* drug release of free Entacapone and optimized Entacapone-NLC.

**Figure 8.**
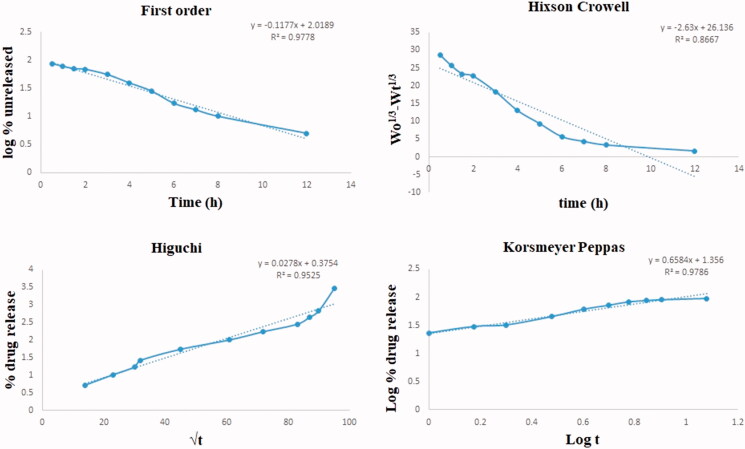
Drug release kinetics of Entacapone-loaded NLCs.

*In vitro* drug release of Entacapone-loaded NLCs showed dual release behavior with initial burst release of the surface-bound drug and then delayed release of the matrix drug through diffusion mechanism. After 12 h approximate 98.82 ± 3.8% drug release was observed from NLCs as compared to the free drug which showed complete release within 2.5 h (Figure 7). This depicts the sustain release nature of Entacapone-loaded NLCs. Moreover, the kinetic models depict that the Entacapone released from NLCs is governed by the Korsmeyer Peppas model, which is indicative of a diffusion-mediated drug release (Figure 8). Similar results were reported by several researchers for NLCs (Khan et al., 2019; Mahmood et al., 2021). A more stringent test was used to distinguish between the mechanisms of drug release. The release data were fitted to the Peppas exponential model, *M*_t_/*M*_∞_ = Ktn, where *M*_t_/*M*_∞_ is the fraction of drug released after time *t*; *k* is the kinetic constant; and *n* is the release exponent which characterizes the drug transport mechanism. It was observed that the *n* value is 0.658 means it is 0.45 < *n* < 0.89 which indicates that it follows non-Fickian transport as reported by Peppas et al. (Ritger & Peppas, 1987; Gurumukhi & Bari, 2021).

#### Accelerated stability studies

In the stability studies before and after the lyophilization of the formulation the effect of temperature was noted. Initial particle size, PDI, and drug content were determined at the time of preparation and then the batch was divided into two equal portions, which were stored under different temperature conditions in a refrigerator 5 °C ± 3 °C for 3 months and at 25 °C ± 2 °C/60% RH ± 5% RH for 3 months. Continuous monitoring of the sample with respect to particle size, PDI, and zeta potential was done for up to 3 months. The obtained results are presented in Table 4. As per the results obtained initially the particle size was 161.2 nm and it was observed that after 3 months of storage slight particle size was increased which might be due to the aggregation and swelling. The result of PDI and zeta potential was 0.444 ± 0.12 and −28 ± 1.1 mV at the time of preparation and 0.497 ± 0.11and −29 ± 1.4 mV after 3 months of storage which seems to be negligible change. whereas the entrapment efficiency was decreased from 82.51 ± 4.2 to 78.32 ± 2.1 which could be due to the leakage of drug from NLCs. Overall the results of stability study depict no significant change during storage of samples for 3 months at both the stability conditions mentioned above. From the data it can be interpreted that optimized Entacapone NLCs were relatively stable under refrigerated conditions.

**Table 4.** Stability study of lyophilized Entacapone-loaded NLCs.

Sampling time	PS (nm)	PDI	Zeta potential	EE %
At the time of preparation	161.2 ± 23.6	0.444 ± 0.12	−28 ± 1.1 mV	82.51 ± 4.2
25 °C ± 2 °C/60% RH ± 5% RH for 3 months				
1 Month	170.3 ± 12.8	0.483 ± 0.22	−28 ± 1.6 mV	81.33 ± 2.6
2 Months	192.7 ± 27.2	0.472 ± 0.18	−27 ± 2.1 mV	82.74 ± 3.1
3 Months	203.5 ± 21.2	0.497 ± 0.11	−29 ± 1.4 mV	78.32 ± 2.1
Storage condition in refrigerator 5 °C ± 3 °C for 3 months				
1 Month	166.3 ± 16.5	0.454 ± 0.23	−26 ± 2.4 mV	80.12 ± 3.2
2 Months	174.6 ± 18.9	0.482 ± 0.21	−27 ± 2.1 mV	79.89 ± 4.2
3 Months	186.4 ± 22.3	0.488 ± 0.19	−27 ± 2.2 mV	70.81 ± 3.6

#### Pharmacokinetic study

**Figure 9.**
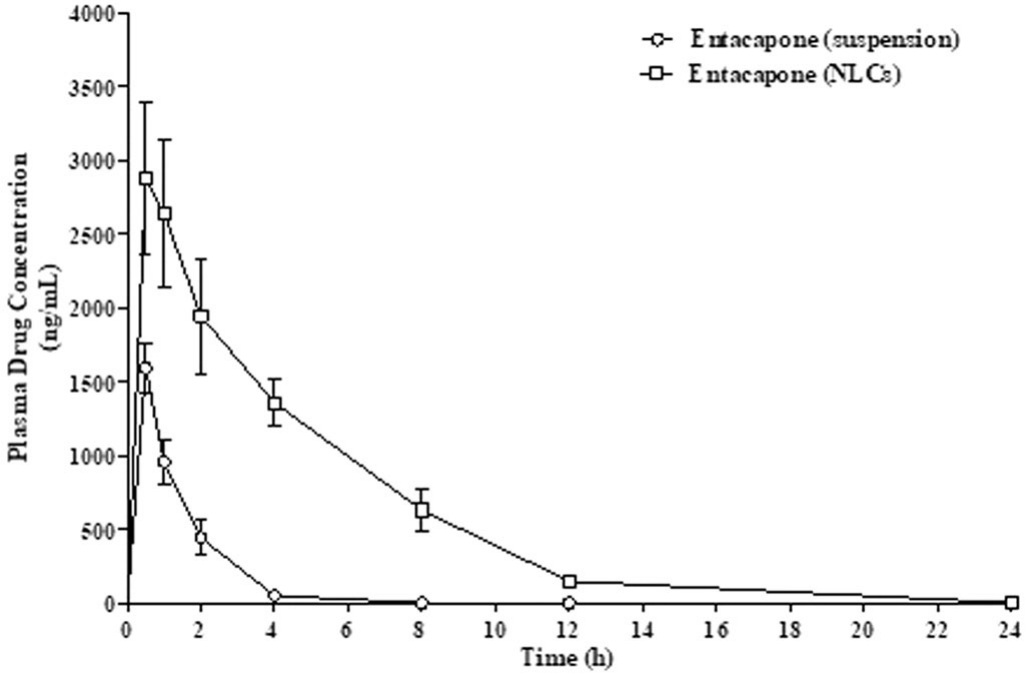
Plasma drug profile of free Entacapone and drug-loaded NLCs in Wistar rats.

After single-dose administration of plain Entacapone and drug-loaded NLCs, the drug was observed in plasma for 24 h above its therapeutic concentration. However, the free drug was cleared from circulation within 1–2.5 h (Figure 9).

The plasma profile shows that *C*_max_ and *T*_max_ of drug-loaded NLCs significantly differed from the free drug. Kel of the free drug was higher than NLCs, indicating an increase in the *t*_1/2_ of the latter. Moreover, the mean residence time (MRT) and AUC_0–∞_ of NLCs are significantly (*p* < .001) increased as compared to a free drug (Table 5).

**Table 5.** Pharmacokinetic parameters of free Entacapone and Entacapone-loaded NLCs.

Parameters	Free Entacapone	Entacapone-NLCs
*C*_max_ (ng/mL)	1592 ± 99	2993 ± 322**
*T*_max_ (h)	0.5 ± 0.08	1.0 ± 0.13*
*t*_1/2 (h)_	0.704 ± 0.01	4.74 ± 0.58***
k_el_'	0.847 ± 0.02	0.14 ± 0.01**
AUC_0–∞_ (ng h)/mL	2227.5 ± 121.6	21,588.3 ± 1067.6***
MRT (h)	1.18 ± 0.12	6.77 ± 0.23***

*Notes*: * , **, *** are Statistically less , moderate and highly significant respectively(*p* < .001) as compared with the plain Entacapone group.

## Conclusion

In the current study, Entacapone-loaded nanostructured lipid carrier was developed by probe sonication method and optimized with QbD approach. The 2^3^ full factorial design was explored to optimize and drive the relationship between the screened critical parameters and the desired responses. The quadratic polynomial model was used to derive significant interaction terms. Moreover, the 2D and 3D surface response graphs were used to interpret the simultaneous impact of all the CQAs on the selected responses. It was found that the concentration of the lipoidal phase plays an important role in particle size, drug entrapment, and drug release from NLCs. SEM studies revealed the non-spherical shape and nano-metric size of the drug-loaded NLCs, which was further confirmed by Malvern Zeta Sizer. Both DSC and XRD studies proved the compatibility of the drug with the lipids used. The *in vitro* release pattern displayed a sustained release pattern of Entacapone from NLCs. In the accelerated stability study, the particle size and zeta potential were stable at room temperature and comparatively more stable in refrigerated conditions. From the *in-vivo* pharmacokinetic results, it can be concluded that the orally administered Entacapone NLCs displayed better bioavailability by enhancing the AUC in plasma drug concentration profile. The nanoscale particle size, enhanced solubilization, prevention of extensive metabolism, and enhanced efficiency will ultimately help to reduce the dose and improve the overall therapy. Hence we conclude that the preparation of Entacapone NLCs can be an appropriate approach for improving the oral bioavailability and pharmacological bioactivity of the poorly water-soluble drugs.
